# 3D Motion Capture May Detect Spatiotemporal Changes in Pre-Reaching Upper Extremity Movements with and without a Real-Time Constraint Condition in Infants with Perinatal Stroke and Cerebral Palsy: A Longitudinal Case Series

**DOI:** 10.3390/s20247312

**Published:** 2020-12-19

**Authors:** Julia Mazzarella, Mike McNally, Daniel Richie, Ajit M. W. Chaudhari, John A. Buford, Xueliang Pan, Jill C. Heathcock

**Affiliations:** 1 Physical Therapy Division, School of Health and Rehabilitation Sciences, College of Medicine, The Ohio State University, 453 W 10th Ave., Columbus, OH 43210, USA; julia.mazzarella@osumc.edu (J.M.); daniel.richie@osumc.edu (D.R.); Ajit.Chaudhari@osumc.edu (A.M.W.C.); john.buford@osumc.edu (J.A.B.); 2 Tampa Bay Rays, 1 Tropicana Dr., St. Petersburg, FL 33705, USA; mcnallym08@gmail.com; 3 Department of Mechanical and Aerospace Engineering, College of Engineering, The Ohio State University, 453 W 10th Ave., Columbus, OH 43210, USA; 4 Department of Biomedical Engineering, College of Engineering, The Ohio State University, 453 W 10th Ave., Columbus, OH 43210, USA; 5 Center for Biostatistics, Department of Biomedical Informatics, College of Medicine, The Ohio State University, 1800 Cannon Drive, Columbus, OH 43210, USA; jeff.pan@osumc.edu

**Keywords:** perinatal stroke, kinematics, upper extremity, cerebral palsy, hemiplegia, constraint

## Abstract

Perinatal stroke (PS), occurring between 20 weeks of gestation and 28 days of life, is a leading cause of hemiplegic cerebral palsy (HCP). Hallmarks of HCP are motor and sensory impairments on one side of the body—especially the arm and hand contralateral to the stroke (involved side). HCP is diagnosed months or years after the original brain injury. One effective early intervention for this population is constraint-induced movement therapy (CIMT), where the uninvolved arm is constrained by a mitt or cast, and therapeutic activities are performed with the involved arm. In this preliminary investigation, we used 3D motion capture to measure the spatiotemporal characteristics of pre-reaching upper extremity movements and any changes that occurred when constraint was applied in a real-time laboratory simulation. Participants were N = 14 full-term infants: N = six infants with typical development; and N = eight infants with PS (N = three infants with PS were later diagnosed with cerebral palsy (CP)) followed longitudinally from 2 to 6 months of age. We aimed to evaluate the feasibility of using 3D motion capture to identify the differences in the spatiotemporal characteristics of the pre-reaching upper extremity movements between the diagnosis group, involved versus uninvolved side, and with versus and without constraint applied in real time. This would be an excellent application of wearable sensors, allowing some of these measurements to be taken in a clinical or home setting.

## 1. Introduction

Perinatal stroke (PS) is caused by interrupted blood flow to the brain between 20 weeks gestation and 28 days of life [1,2]. PS affects ~24.7/100,000 live births in the US annually [3]. Common impairments as a result of the stroke include delays and impairments in motor, sensory, cognitive, speech, and hearing abilities [4]. Many infants with PS do not demonstrate clinical signs right away, unless they develop seizures (resulting in a referral for brain imaging), and are often diagnosed once motor asymmetries appear—months, if not years, after the original brain injury [5]. PS is the leading cause of hemiplegic cerebral palsy (HCP), in which the arm, leg, and trunk on one side of the body are more affected (involved side) by motor and sensory impairments than the other side (uninvolved side). HCP leads to the impaired or delayed development of reach and grasp, affecting the child’s upper extremity skills. The early detection of these impairments in at-risk infants is crucial in order to provide appropriate referral and begin rehabilitation interventions.

The diagnosis of cerebral palsy (CP) is occurring at earlier ages, due in part to the recently published guidelines and implementation of early diagnosis criteria [6,7,8]. Magnetic resonance imaging (MRI) is sometimes used in the diagnostic process, but a diagnosis of CP is not given without an observation of motor impairment. Current tools used to aid in diagnosis are based on reflexes and motor skill performance, such as the Hammersmith Infant Neurological Exam (HINE) and the Test of Infant Motor Performance (TIMP) [6]. The early identification of CP is enhanced using the General Movement Assessment (GMA), which uses a gestalt observation of movement quantity and quality [9,10]. The observations of abnormal movement patterns using the GMA as early as 10 weeks of age have been shown to have strong predictive validity for a later CP diagnosis [11]. Combinations of these tools have been used to improve diagnostic predictive ability, but there remains substantial room for improvement, given the time between the injury to the central nervous system (CNS) and diagnosis of motor impairment. There is a need for objective measures of motor impairment in these infants for earlier, definitive diagnosis and a better understanding of the underlying pattern of impaired coordination which is a result of an injured, developing nervous system. 3D motion capture may offer a technology-based solution to detect the asymmetries and abnormal movement patterns in at-risk infants [12,13]. By measuring kinematic characteristics of pre-reaching movements, we might be able to use 3D motion capture to detect motor impairment prior to the emergence of clinical signs like an early arm preference or delayed onset of reaching. Such a tool would aid in the early identification of the underlying movement impairment and provide impairment-level targets for rehabilitation. Importantly, delivering targeted interventions earlier in development leads to better motor outcomes for children with CP [14].

Previous studies using 3D motion capture have identified the spatial and temporal parameters of reaching and pre-reaching or spontaneous movements in infants with typical development (TD) and in infants at high risk for CP [15,16,17,18,19,20,21,22,23]. A reaching movement is a movement of the hand that ends in a hand–toy contact, typically in a midline position, whereas a pre-reaching or spontaneous movement is any movement that does not meet the definition of a reach [17]. In research studies, these pre-reaching movements are often defined as movements toward a toy and against gravity [13,17]. Most typically developing infants demonstrate an onset of reaching in a midline position around 3–5 months of age [21]. The developmental trajectory of reaching movements includes an (i) increased frequency of successful hand–toy contacts with a decrease in spontaneous or pre-reaching movements; (ii) straighter hand path measured by a lower straightness ratio, which means the length of the hand path is getting shorter relative to the distance between the start and end point of the reach [23]; and (iii) faster movement speed early in development with a decrease in movement speed with the onset of reaching, in order to perform a more accurate movement [21]. Infants with CP show delays in the onset of reaching, less coordinated reaches (a smaller number of reaches, longer straightness ratio, faster speeds, and an increased number of movement units), and often an early arm preference [16,24,25], whereas TD infants will demonstrate a fluctuating arm preference [15,20,23,26].

Constraint-induced movement therapy (CIMT) is a promising treatment option for infants with HCP. CIMT involves constraining the uninvolved arm, using a cast, a mitt, or by a therapist holding the arm, in order to provide therapy and encourage as much meaningful use and repetition of the involved arm as possible. Pre- and post-CIMT treatment outcomes are commonly measured, and have demonstrated positive treatment effects including improvements in kinematics [27,28,29,30,31,32,33]. Recent studies have also shown an increased overall use of the involved limb during CIMT [34]. The effects of constraint in real time on spatial and temporal kinematics of upper extremity movements, however, are unknown. Monitoring the kinematic characteristics of upper extremity movements during the intervention could provide important feedback to therapists on the effectiveness of the intervention and allow for the moderation of the intervention to increase desired behaviors. It could also help to understand the underlying motor impairment and detect how CIMT may impact recovery and development for these infants. Therefore, identifying a tool, such as 3D motion capture, that can track these behaviors in real time, could greatly improve the therapeutic impact and outcomes for infants with PS and CP.

In this preliminary investigation, our objective was to answer two primary questions that will ultimately serve to guide future research. (i) Can 3D motion capture be used to detect differences in pre-reaching and reaching behaviors, in terms of the timing and coordination of pre-reaching upper extremity movements and frequency of reaches, between infants with TD, PS, and CP? If so, what are the differences, and do they change with side (involved versus uninvolved) and age? (ii) Can 3D motion capture detect changes in pre-reaching and reaching behaviors with and without the constraint of an arm? If so, what are the changes, and are they differ with diagnosis, side, and age? The purpose of this study was to generate specific hypotheses to guide future research on the use of 3D motion capture and wearable sensors to objectively track changes in upper extremity gross motor skills in infants with PS and CP, either for the early detection of impairment, or to monitor an intervention. Moreover, we aimed to identify which specific kinematic variables could serve as biomarkers for typical versus impaired upper extremity motor development in infants with PS and CP.

## 2. Materials and Methods

### 2.1. Participants

There were N = 14 full-term (>37 weeks gestational age) infants enrolled: 6 with TD, 5 with PS and no diagnosis of CP, and 3 with PS who later received a diagnosis of CP. PS was confirmed with MRI by a radiologist. The CP diagnosis, given to 3 of the infants with PS, was a clinical diagnosis made by a physician, which we confirmed via chart review at an 18-month follow up. The infants with PS were recruited from Nationwide Children’s Hospital in Columbus, OH, and by word of mouth. The infants with TD were recruited from the Columbus, OH area through word of mouth. Some of the infants included in this preliminary analysis were part of a larger study on upper extremity development in infants with PS. Exclusion criteria for both groups included genetic disorders, and orthopedic or visual impairments that could affect reaching behaviors. In order to capture early arm movements, prior to the onset of reaching, infants came to the lab for their first data collection at around 2 months of age (range 60–84 days old); 75.5 ± 9.3 days for the TD group, 70.4 ± 9.7 days for the PS group, and 67.5 ± 10.3 days for the CP group. There was no significant difference in age between the groups (F (2) = 0.96, p = 0.41). Parents were informed of the risks and potential benefits of their child participating in this study. Parental permission was obtained prior to the start of data collection. The Ohio State University Institutional Review Board (BUCK IRB# 2008H0197, 2019N0012) and the Nationwide Children’s Hospital Institutional Review Board (NCH IRB# IRB08-00292) approved this study and the data collection from human subjects that was performed therein.

### 2.2. Procedure

**Figure 1:**
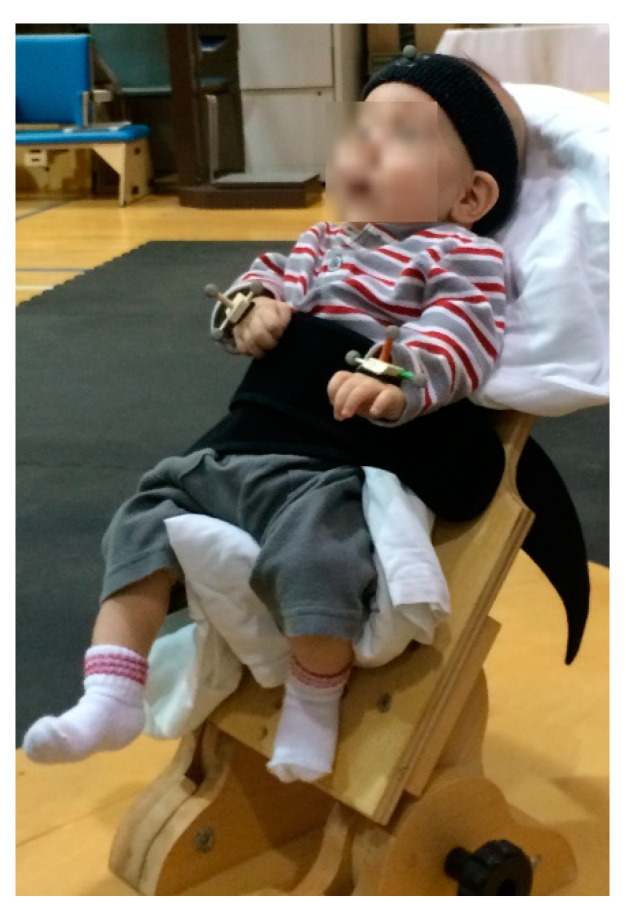
Infant seated in the custom chair and wearing retroreflective markers for 3D motion capture.

In this longitudinal study, infants came to the lab monthly for 5 sessions over a 5-month period, starting at 2 months of age, in order to capture the window of pre-reaching and reach onset in typical development. The infants’ movements were captured using a 10-camera Vicon motion capture system (Vicon Motion Systems Ltd., Oxford, UK) [35]. The infants were seated in a custom-made chair reclined 30° from vertical with a wide strap securing their torso against the back of the chair, while still allowing for free arm movement (Figure 1) [17]. Eight-millimeter diameter retroreflective markers were placed on the infant: 3 on each hand, 1 on the forehead, 3 on the chair, and 1 on the toy. At each session, movement was recorded at 120 Hz for nine 30 s trials: 3 with both hands free (bilateral; Figure 1), and 3 each with either arm constrained by the experimenter holding it against the infant’s side (constraint). The trial length was selected based on previous 3D motion capture studies of pre-reaching arm movements [13,17,36]. A toy was presented in front of the infant within arm’s reach at midline and shoulder height to stimulate upper extremity movement [13,17]. The 3D position for each marker was calculated in Vicon Nexus 1.8.5 and low-pass filtered with a 4th order zero lag Butterworth filter, with a cutoff frequency of 4 Hz [17,18,19,20]. The 3D linear positions and speed were calculated for each marker. 

The spatial variables calculated for pre-reaching arm movements were movement length (length of straight line from start point to end point of a movement in mm), length of the hand path (total distance travelled by the hand from start to end of the movement in mm), and straightness ratio (ratio of length of hand path to movement length). The temporal variables calculated were the movement speed (average velocity of the movement, calculated by movement length over time in mm/s), movement frequency (total number of movements in a trial over time in minutes), and reach frequency (total number of reaches in trial over time in minutes). The variables selected were all reliable measures of upper extremity coordination in pediatric populations [16,17,20,21,23,24,26].

Each dependent variable reported was calculated as a per-movement average. In this study, we applied the definition developed by Bhat and Galloway (2006) for a “movement”: a hand displacement of at least 30 mm in one direction, the end of which was indicated by a reversal of direction measuring 15 mm or greater in length; and a “reach”: a movement that results in the hand making contact with the toy [17,26]. Movements and reaches were identified using a custom MATLAB program (The Mathworks Inc., Natick, Massachusetts) [37]. Reaches were confirmed with the observation of video recordings of the trials. The calculations for the dependent variables were performed using MATLAB. Each trial was quality checked by viewing a graph of the trajectory of each hand. The start and stop points for each movement were labelled on the graphs and confirmed or corrected by an experimenter. The quality check process ensured that no obvious artifacts were included in the statistical analysis.

### 2.3. Statistical Analysis

Means and standard deviations of each variable were calculated for analysis by age for each group, trial type, and side from multiple repeated trials for each participant at each time. Linear mixed models for repeated measures were used to estimate the changes of each dependent variable over time by group (CP, PS, TD). This statistical model was structured to account for the association of repeated measures both over time and from the two sides (arms) of the same participant at each time point. In the analysis, age was treated as a categorical variable by months of age. For infants with bilateral injuries based on the MRI, where no side was classified as having greater involvement, both upper extremities were categorized as potentially involved in the analysis. Due to the small sample size, no formal statistical tests were conducted to compare the changes from 2 to 6 months among different diagnosis groups. Exploratory analyses were conducted to estimate the changes of the same measure over time for each side by different groups. In addition, the estimated mean and standard deviation at 2-month and 6-month time points for all dependent variables were summarized for each group to be used for the design of future studies. Variability was represented by the standard deviation of the mean. SAS version 9.4 (The SAS Institute, Cary, NC, USA) [38] was used to conduct the statistical analysis.

## 3. Results

In this study, we successfully collected longitudinal 3D motion capture data on 14 infants with and without PS and CP, prior to and during the emergence of reaching. A total of 642 trials containing 9784 unique movements were included in the statistical analysis, with at least one usable trial of each type per session per participant. We will first give a description of the overall group differences from the first to the final data collection. In the subsequent sections, we will describe the changes between and within diagnosis groups in terms of the side (involved versus uninvolved), age, and constraint versus no constraint (bilateral) in more detail.

To address our first question, whether 3D motion capture can detect group differences in the timing and coordination of pre-reaching and reaching movements, we observed an overall change in the means from 2 to 6 months for both spatial and temporal kinematic variables, indicative of possible group–age interactions (Table 1). At 2 months of age, all three groups had similar means for all kinematic variables, in that they were all within one standard deviation of the TD group. At 6 months of age, there were visible differences between the group means for many of the variables. There was some noticeable variability in the means from 3 to 5 months, with trends becoming most apparent at 5 and 6 months.

In terms of spatial variables, the infants with PS and TD both demonstrated an increase in the mean movement length and path length from 2 to 6 months but the CP group did not: for the infants with PS, both the movement and path length means were more than one standard deviation longer at 6 months than at 2 months (62.7 ± 13.1 to 83.9 ± 16.8, 116 ± 20.4 to 156 ± 32.1, respectively). Conversely, the infants with CP demonstrated *decreased mean movement and path lengths* at 6 months compared to 2 months. Not surprisingly, since movement and path length both changed in the same direction within groups, the straightness ratio did not change noticeably in any of the groups from 2 to 6 months.

In terms of the temporal variables, the infants with CP showed a slightly slower mean movement speed of 116 mm/s, although still within one standard deviation of the other two at 2 months. At 6 months, the infants with CP increased their mean movement speed to 154 mm/s, comparable to the 2-month means of the other two groups; however, the infants with PS and TD increased their mean movement speeds from 151 and 138 to 203 and 273 mm/s by 6 months, respectively. For movement frequency, all groups had similar mean frequencies at 2 months, and the infants with PS and TD appeared to maintain their mean frequencies around 18 movements/min from 2 to 6 months, while the infants with CP decreased to a mean of just 6.3 movements/min at 6 months, which is within two standard deviations below the means of the other two groups. Finally, all three groups had a mean reach frequency close to 0 reaches/min at 2 months. The infants with CP remained near 0 reaches/min at 6 months, while the infants with PS had a mean of 3.46 reaches/min and the infants with TD a mean of 3.63 reaches/min, indicative of the typical onset of reaching.

To address our second question, whether 3D motion capture detected changes with versus without constraint, we found that this varied based on the diagnosis group, age, and side (involved versus uninvolved). We will describe the differences that were observed for each variable in the proceeding sections.

### 3.1. Spatial Variables

**Figure 2:**
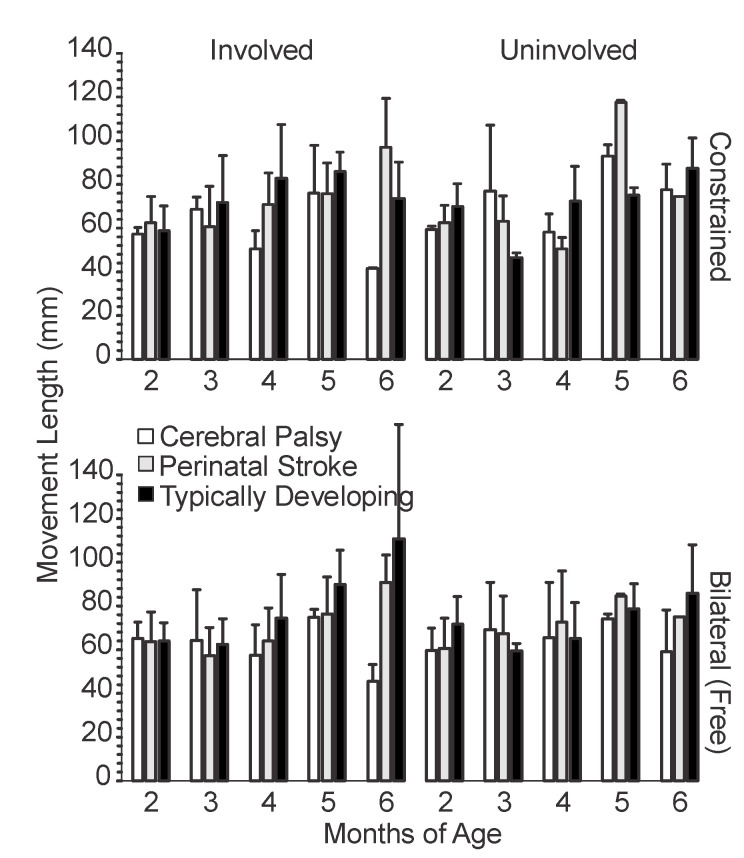
Mean movement length by age, side and condition. For typically developing infants, the left arm is plotted alongside the involved limb and the right arm is plotted alongside the uninvolved limb. Error bars show 1 standard deviation.

**Figure 3:**
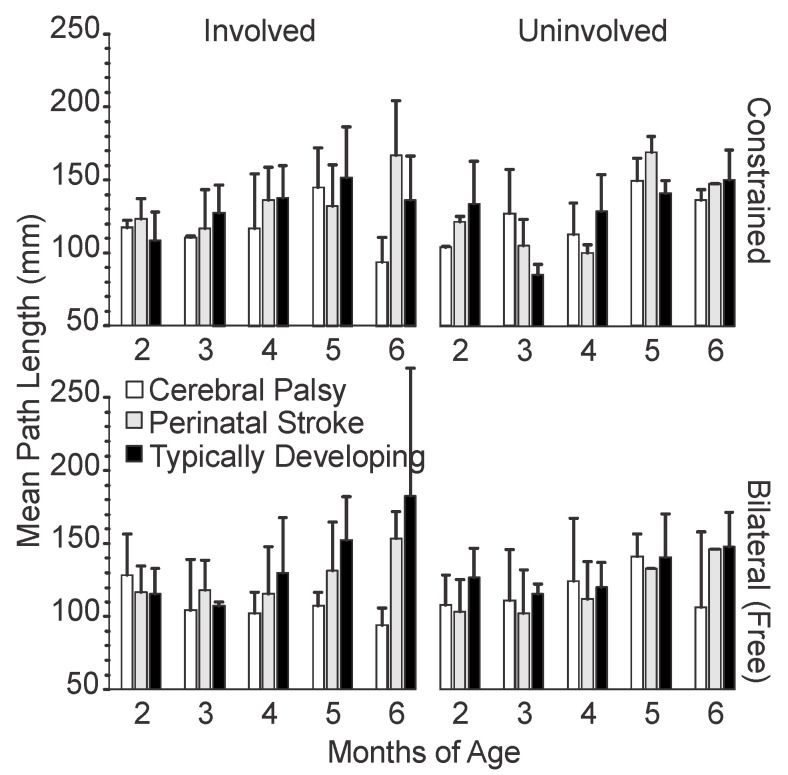
Mean hand path length by age, side, and condition. For typically developing infants, the left arm is plotted alongside the involved limb and the right arm is plotted alongside the uninvolved limb. Error bars show 1 standard deviation.

**Figure 4:**
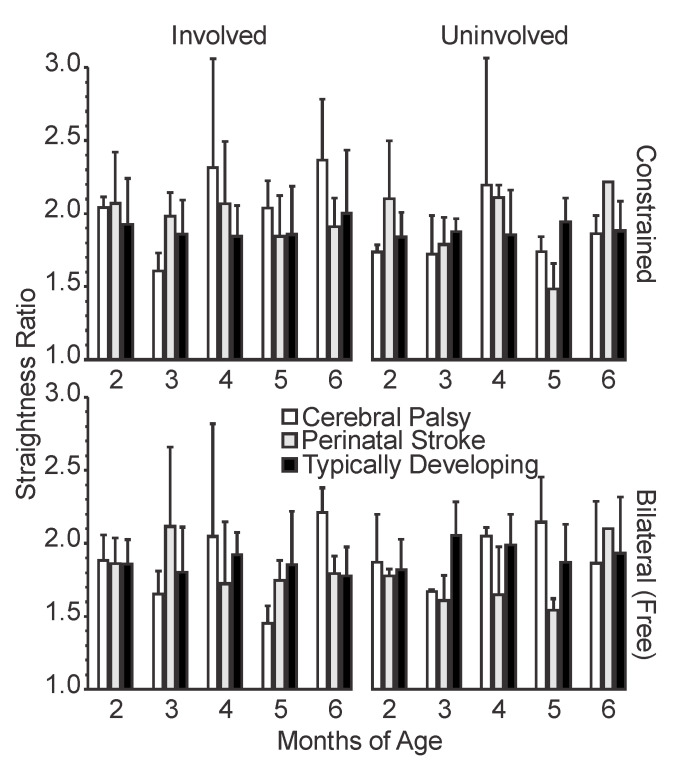
Mean straightness ratio by age, side, and condition. For typically developing infants, the left arm is plotted alongside the involved limb and the right arm is plotted alongside the uninvolved limb. Error bars show 1 standard deviation.

We measured three spatial variables of pre-reaching movement: movement length (Figure 2), length of hand path (Figure 3), and straightness ratio (Figure 4), as defined in Section 2.2. With regards to our second question, the infants with CP did demonstrate some consistent differences in their involved side with constraint versus bilateral, with a longer mean path length and larger mean straightness ratio in the constraint versus bilateral condition, which was not observed for the PS group or for any group with movement length.

#### 3.1.1. Movement Length 

Infants in all three groups (TD, PS, CP) had an average movement length around 60 mm at 2 months for both sides in the constraint and bilateral conditions (Figure 2). The changes with age differed between diagnosis groups. The infants with TD and PS both increased mean movement length over time, while the infants with CP did not, and even showed a trend for shorter movement length in the involved side with age, which addresses our first question.

The infants with TD demonstrated increased movement length with age, more so in the bilateral condition than the constraint condition. In the constraint condition, there was more variability, but still a gross increase in mean movement length from 2 to 6 months. 

The infants with PS showed similar changes with age in the bilateral condition as the infants with TD, although the mean movement length for the uninvolved side in the bilateral condition did decrease from 5 to 6 months. As with the infants with TD, infants with PS had more variability with age in the constraint condition, particularly with the uninvolved limb, which showed a decrease at 4 months, an increase at 5 months, followed by another decrease at 6 months. The overall change from 2 to 6 months was an increase in mean movement length for both limbs and both conditions. At 6 months, the infants with PS had mean movement lengths about 20 mm less in the uninvolved limb versus the involved limb in both the constraint and bilateral conditions.

The infants with CP followed a variable trend in both sides and conditions similar to the uninvolved limb of the infants with PS in the constraint condition. There were peaks in the movement length at 3 and 5 months for all conditions for the infants with CP and decreases at 4 and 6 months. The movement length for infants with CP was about the same at 2 months as it was at 6 months for the uninvolved side in the bilateral condition. For the involved side of the infants with CP in both conditions, the movement length was shorter at 4 and 6 months than at 2 months. Only the uninvolved side in the constraint condition for infants with CP showed increased movement length from 2 months to 6 months. In all conditions, the longest movement length for infants with CP occurred at 5 months.

#### 3.1.2. Length of Hand Path

In human movement, the length of the hand path (Figure 3) can never be shorter than the movement length (Figure 2). In this study, all groups had similar mean path lengths at 2 months. The infants with TD and PS overall had increased hand path length with age, corresponding to the increased mean movement lengths they demonstrated. The infants with CP showed a more variable pattern again, with shorter path lengths on the involved side at 6 months versus 2 months. At the age of 6 months, the path lengths for CP, PS and TD were: 103 ± 26.3, 156 ± 32.1 and 155 ± 47.2 mm respectively, demonstrating a difference of more than one standard deviation between the infants with CP and TD.

With constraint, the infants with PS and TD both showed more variation over time compared to bilateral trials. The infants with PS showed a decrease from 2 to 4 months, followed by a sharp spike from 4 to 5 months, followed by a decrease from 5 to 6 months in the uninvolved side constraint condition, which was similar to the trend seen with movement length. The infants with PS showed an inverse pattern with the constraint of the potentially involved limb, increasing from 3 to 4 months, decreasing from 4 to 5 months, then increasing again from 5 to 6 months. The infants with CP did not show clear differences between the constraint and bilateral conditions for mean path length, although they did demonstrate a shorter mean path length in their involved side, particularly in the bilateral condition, after 2 months.

#### 3.1.3. Straightness Ratio

Infants in all three groups started out with a similar mean straightness ratio at 2 months (Figure 4). Noticeably, the infants with CP and PS showed greater variation in the straightness ratio between sides and conditions, whereas the infants with TD maintained a relatively constant straightness ratio for both sides and conditions over time.

The infants with CP showed decreased straightness ratios at 3 and 5 months for both sides and conditions. Interestingly, at 5 months, the infants with CP had a lower straightness on their involved side versus their uninvolved side in the bilateral condition, indicating straighter movements with the involved arm. The infants with CP also had a higher straightness ratio for the involved side in the constraint condition compared to bilateral, while the uninvolved side showed the opposite pattern. At 6 months, the infants with CP had a higher straightness ratio with the involved side in both conditions compared to the uninvolved side, which indicated less straight movements on the involved side. 

The infants with PS showed a slight trend for decreased straightness ratio in the involved side from 2 to 6 months, with some variability. The uninvolved side followed a similar trend, although there was a sharp increase for the uninvolved side in both conditions from 5 to 6 months. For the infants with PS, the uninvolved side in the bilateral condition showed the lowest straightness ratio at all time points, except at 6 months, indicating the straightest movements in that condition. Additionally, at most time points the straightness ratio for infants with PS is higher for the constraint condition than the bilateral condition for both involved and uninvolved sides, indicating straighter movements in the bilateral condition.

The infants with TD did not demonstrate notable differences in straightness ratio with condition, side, or age.

### 3.2. Temporal Variables

**Figure 5:**
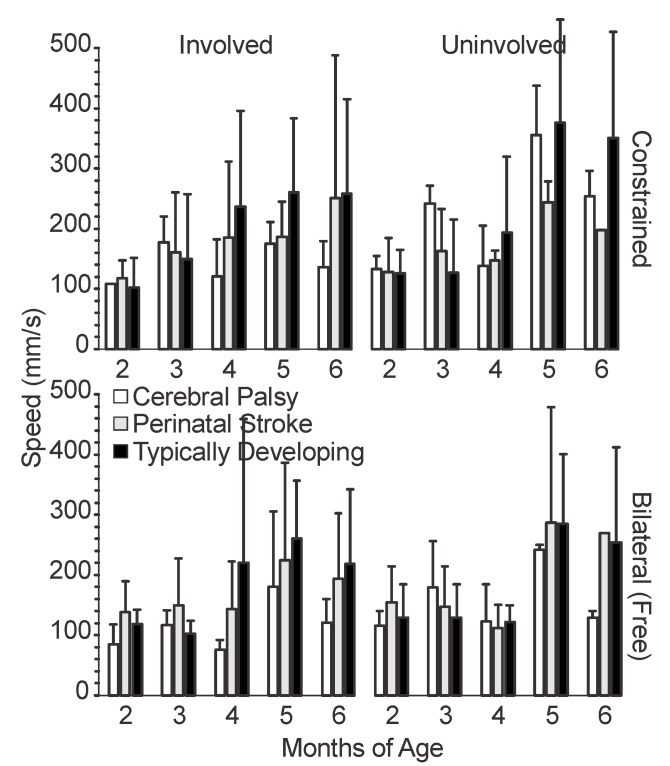
Mean movement speed by age, side, and condition. For typically developing infants, the left arm is plotted alongside the involved limb and the right arm is plotted alongside the uninvolved limb. Error bars show 1 standard deviation.

**Figure 6:**
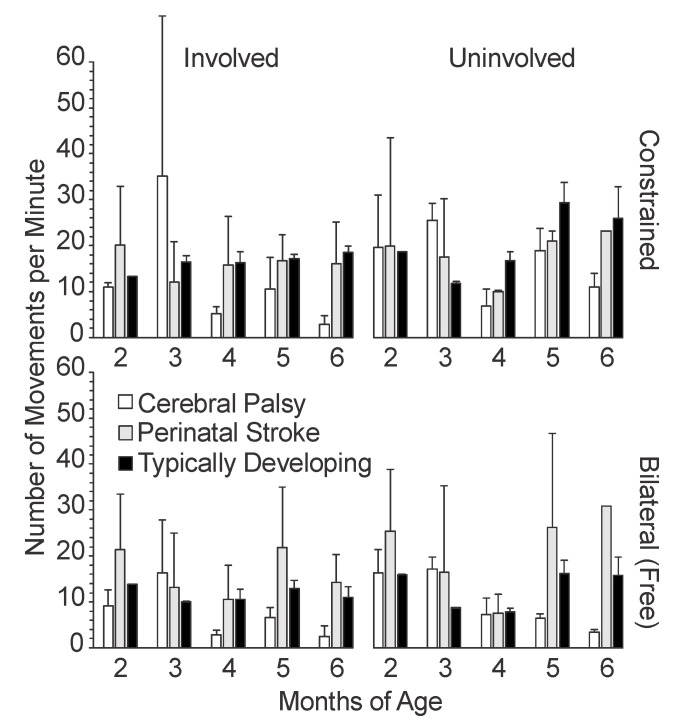
Mean movement frequency by age, side, and condition. For typically developing infants, the left arm is plotted alongside the involved limb and the right arm is plotted alongside the uninvolved limb. Error bars show 1 standard deviation.

**Figure 7:**
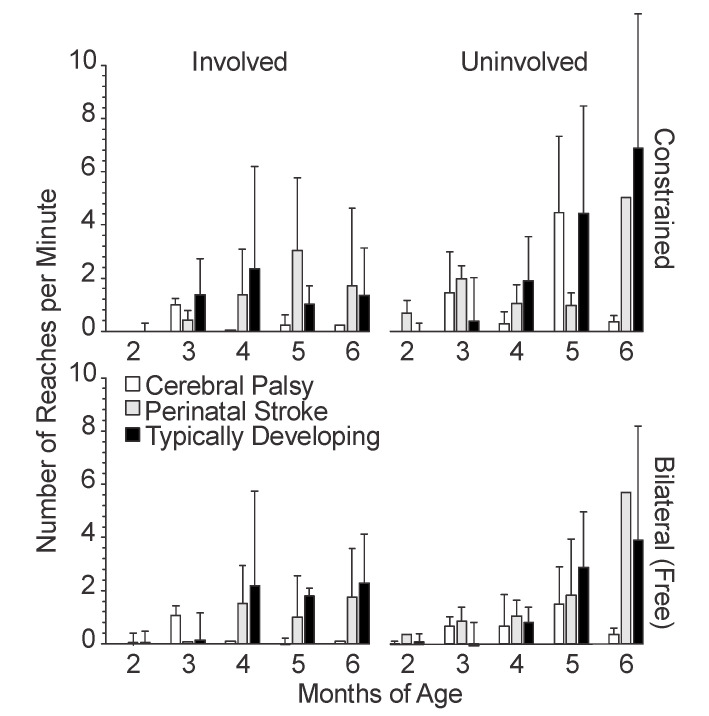
Mean reach frequency by age, side, and condition. For typically developing infants, the left arm is plotted alongside the involved limb and the right arm is plotted alongside the uninvolved limb. Error bars show 1 standard deviation.

We measured three temporal variables of upper extremity movement: movement speed (Figure 5), movement frequency (Figure 6), and reach frequency (Figure 7), as defined in Section 2.2.

#### 3.2.1. Movement Speed

All three groups demonstrated an increase in movement speed in both sides and both conditions from 2 to 6 months (Figure 5). There were potential differences between groups at 2 months; the mean for the involved side of the infants with CP was lower than those for the infants with PS and TD. At 6 months, the mean movement speed for the uninvolved side in the constraint condition for infants with CP was similar to the means of infants with TD and PS, but the means for the involved side in both conditions, and the uninvolved side in the bilateral condition, were substantially lower. Additionally, all three groups showed a decrease in the movement speed from 5 to 6 months for both sides and conditions, with the exception of the involved side of the infants with PS, and the right hand of the infants with TD, in the constraint condition.

The infants with CP showed peaks in their movement speed at 3 and 5 months and decreases at 4 and 6 months for both sides and conditions. The infants with CP appeared to move at faster speeds, on average, with their uninvolved limb compared to the involved limb. Additionally, they moved at faster speeds, on average, in the constraint conditions, compared to the bilateral conditions. The slowest movement speeds were consistently in the involved side in the bilateral condition at all time points for the infants with CP.

The infants with PS demonstrated a general increase in movement speed with age in both conditions and sides, although there was a decrease noted at 4 and 6 months for all except the involved side in the constraint condition. There was no clear difference in movement speed between sides or conditions for the infants with PS, although at 5 and 6 months, the mean movement speeds were fastest with the uninvolved side in the bilateral condition.

The infants with TD demonstrated a sharp increase in mean movement speed from 4 to 5 months with the left side, and from 3 to 5 months with the right side. From 5 to 6 months, we saw a decrease in the mean movement speed in all but the right side in the constraint condition. The infants with TD also had faster mean speeds in the constraint condition compared to the bilateral for the left side at 4 to 6 months, but not with the right side.

#### 3.2.2. Movement Frequency

The infants with CP and TD both had higher mean movement frequencies with constraint compared to the bilateral conditions (Figure 6). The infants with PS showed no clear difference in the mean movement frequency between conditions, although they did have a higher mean movement frequency with the uninvolved side compared to the involved side at 6 months. The infants with TD and PS both showed a minor trend for increased movement frequency between 4 to 6 months, although this increase was more pronounced in the infants with PS, particularly in the uninvolved limb. Meanwhile, the infants with CP had a sharp decrease in the mean movement frequency from 3 to 4 months, which increased slightly at 5 months, then decreased again at 6 months. Interestingly, the infants with CP started out with similar mean movement frequencies as the infants with TD at 2 months, then increased to much higher mean frequencies at 3 months in the constraint condition, then decreased sharply to frequencies below those of the infants with PS and TD at 4 to 6 months. The infants with PS started at higher movement frequencies than the two other groups, but then showed mean movement frequencies similar to the infants with TD for the remaining 4 months.

#### 3.2.3. Reach Frequency

Infants with TD and PS both showed an overall increase in the reach frequency with age, particularly after 3 months (Figure 7). The infants with CP mostly did not reach with their involved side, except at 3 months and 5 months, and only in the constraint condition. The infants with CP did show an increase in the mean reach frequency of the uninvolved side at 3 and 5 months, more so in the constraint condition than bilateral, however, there were no reaches at 6 months. The infants with PS demonstrated a fairly steady increase in the mean reach frequency with age, albeit with some fluctuation. At 6 months, the involved side of the infants with PS had a much lower mean reach frequency than the uninvolved side. The infants with TD showed a steady increase from 3 to 6 months with the left side, with the highest mean reach frequency in the left side constraint condition at 6 months. There was an increase in mean reach frequency from 2 to 4 months on the right side in both conditions for infants with TD, but then a plateau from 4 to 6 months.

## 4. Discussion

In this study, we demonstrated that the 3D motion capture may feasibly be used to measure objective changes in pre-reaching upper extremity movements in infants with TD and infants with neuromotor impairment, namely PS and CP. We successfully measured three spatial and three temporal variables of upper extremity movement over a 5-month period in 14 infants with TD, PS, and CP. Moreover, we observed trends in the data that generated several hypotheses, which can be used for the design of further investigation. The main trends that we identified were related to differences with diagnosis, changes with the real-time constraint of one arm, and changes over time with age and development. In the subsequent sections, we will discuss the specific hypotheses that we generated from these data, which can be applied to future research, and support for those hypotheses based on previous research.

### 4.1. Hypothesis 1: 3D Motion Capture Can Be Used to Detect Differences in Kinematic Characteristics of Pre-Reaching Movements between a Diagnosis of CP and TD, Particularly through an Interaction Effect with Age

We observed trends for an age–diagnosis interaction for many of the dependent variables measured. Most noticeable was the variability seen in the infants with CP and PS compared to infants with TD. The infants with TD showed apparently linear trends with age that were consistent with previous research, such as increased reach frequency, increased movement length, and increased movement speed [17,20,21,36]. The infants with PS largely followed the trends of typical development, however, with more fluctuation from month to month than the TD group. In contrast, the infants with CP had trends in the opposite direction of typical development for multiple variables, including decreased movement frequency, decreased movement length, and no change in reach frequency. Furthermore, their month-to-month performance was more variable. These differences might be indicative of motor impairment as a result of CNS injury as these infants all had a later diagnosis of CP. The results from this study suggest that it might be possible to use 3D motion capture to differentiate between CP and no CP in infants with PS and between CP and TD, based on kinematic measures of pre-reaching movements.

### 4.2. Hypothesis 2: 3D Motion Capture Can Be Used to Detect Asymmetries in Kinematic Characteristics of Pre-Reaching Movements between the Involved and Uninvolved Side in Infants with PS and CP

We found preliminary evidence that 3D motion capture could be used to detect asymmetry in pre-reaching movements in infants with PS and CP for certain spatiotemporal variables. In our analysis, the infants with CP demonstrated largely decreased movement length, path length, movement speed, movement frequency and reach frequency in their involved side versus uninvolved side. Previous research has found that infants with HCP often begin to present with an increased asymmetry in upper extremity use with development, but also that many of them demonstrate some impairment in the uninvolved limb as well, in terms of the speed and accuracy of movement [24]. In this study, the infants with TD and PS showed a steady increase in the movement speed and reach frequency with age, consistent with typical development [21,39]. The infants with CP showed only a slight increase in movement speed and more notably, only reached at 3 and 5 months, predominately with the uninvolved arm. The slower movement speed and lack of reaches in the infants with CP might indicate impairment in the involved upper extremity, and to a lesser extent, the uninvolved upper extremity, as infants with HCP often demonstrate some impairment or delays in both upper extremities [40].

The infants with PS did not show consistent differences between the potentially involved and uninvolved upper extremities, but based on our results, a larger sample size might reveal a larger straightness ratio, slower movement speed, and decreased movement and reach frequencies in the potentially involved limb, particularly at 5 and 6 months of age. The infants with PS who did not later receive a diagnosis of CP might be demonstrating typical upper extremity development, given that their means were similar to those of the infants with TD. We might speculate, however, that there were some signs that the infants with PS were not following the same pattern of development as the infants with TD, based on the increased fluctuation in the means from month to month. The subtle differences between infants with PS and TD for some kinematic variables of pre-reaching movements (movement frequency, reach frequency, movement speed, straightness ratio), particularly with the involved limb, might represent sub-clinical signs of motor impairment, or it might just be due to the small sample size. Further research is needed to make a definitive conclusion about the results of the PS group.

### 4.3. Hypothesis 3: 3D Motion Capture Can Be Used to Detect and Monitor Changes in Spatial and Temporal Characteristics of Pre-Reaching Upper Extremity Movements with Constraint Versus without Constraint in Infants with TD and CP

Our preliminary analysis indicated that real-time changes with constraint could be observed in the infants with CP and infants with TD for multiple variables of upper extremity movement. These changes were most apparent in the temporal variables. Infants with CP and TD both demonstrated increased movement speed, movement frequency, and reach frequency in the constraint condition compared to the bilateral. For the infants with TD, the difference between the conditions became more apparent at 4–6 months of age, which was consistent with the onset of reach development, usually occurring between 3 and 5 months of age [21]. The infants with CP also demonstrated less precise movement with constraint on their involved side, indicated by a longer hand path length and a larger straightness ratio. Previous studies found the reverse trend for typical development, where the infants demonstrated straighter movements, with a smaller straightness ratio, as they got older, highlighting another possible indication of motor impairment in the infants with CP in this study [23]. Based on our preliminary results, it is possible that constraint increases the use of the free arm in real time in both infants with TD and CP. Our preliminary results also indicate that the infants with CP might show less precise movements in their involved arm with constraint in real time, likely due to poorer coordination and overall development in that arm as a result of both motor impairment and neglect [4,25,41].

### 4.4. Limitations

This study has limitations due to its small sample size because it is a preliminary study. Insights that the data from this initial exploration provide are valuable for generating hypotheses for future investigation. We attempted to not overstate our results and present them in a way that was consistent with the study design. In order to draw any firm conclusions from the results of this study, it needs to be replicated with a larger cohort and sufficient power. In this study, we generated hypotheses about many independent and dependent variables by observing trends in the means from the data we collected. While we observed many plausible and hypothesis-generating trends, there is a risk with the small sample size that results could be skewed by a single participant. Based on our results, we observed the difference in movement length between infants with CP and TD to be at least one standard deviation with an 85% lower boundary of about 0.7. A sample size of 34 per group in a future study could provide at least 80% power to detect such a difference.

Additionally, in this study, we used a 10-camera, 3D motion capture system (Vicon Motion Systems, Ltd., Oxford, UK). It is possible that the cost and access to a 3D motion capture system could pose a barrier to the replication or widespread application of our results. The ongoing development of more inexpensive and portable wearable sensors that can calculate similar variables as 3D motion capture will hopefully make the collection of kinematic data much more accessible and widely applicable. There has been considerable progress in the methods and technology for wearable use in infants with most of the information on physical activity and 24 h monitoring [42,43,44,45,46,47].

Another limitation of this study is the relatively large number of biomechanical variables involved and their interdependence with each other. For example, the straightness ratio is a combination of movement length and path length. Movement speed and movement length are inextricably related. Thus, in a future study, care would need to be exercised for appropriate statistical comparisons between the correlated variables. Furthermore, the relatively large number of measurements that is possible increases the risk of false positives.

### 4.5. Application and Future Directions

A major takeaway from this study is that 3D motion capture may be feasible for the longitudinal tracking of pre-reaching and reaching behavior in infants with TD, PS, and CP. It is possible that further research could identify specific kinematic variables that could be used as biomarkers in infants with neuromotor impairment [12]. The benefit of a biomarker is that it can be monitored with intervention and over time in at-risk infants, in order to measure progress and inform precise rehabilitation to attain optimum health outcomes. 3D motion capture or future wearable sensors could be used for the monitoring of spatial and temporal characteristics of pre-reaching movements. The ability to measure objective changes in the spatial and temporal characteristics of upper extremity movement could be extremely beneficial to a clinician who is using CIMT or another intensive upper extremity intervention for a child with HCP. In the future, we would like to replicate this study with a larger cohort, as well as with wearable sensors.

Wearable sensors, such as inertial measurement unit (IMU) sensors, are becoming increasingly useful for measuring the kinematics of human movement, as they are a small, portable alternative to 3D motion capture. Previous studies in adults have found IMU sensors to perform comparably to the Vicon 3D motion capture system, in terms of measuring joint angles [48,49]. IMU sensors have been successfully applied to measure infant leg movement, mostly in terms of movement frequency [43,44,45,46]. Of note, most of these studies collect data on leg movements that are mostly single-plane and sampled over a duration of one or several days. These are simpler than the multi-dimensional upper extremity movements we measured and are sampled from a much larger data set. Despite the greater complexity of tracking infant upper extremity movements, rapidly emerging wearable technologies using IMU sensors show promise in this application. Notably, IMU sensor systems have been evaluated for their feasibility to measure infant limb kinematics, and with the collection of movement data over a shorter duration, like 60 s [42,47,50]. The variables measured in these kinematic studies using IMUs include the frequency, duration, and acceleration of movements in both the upper and lower limbs, similar to some of the variables we measured in this study [42,47,50]. Some of these recent infant IMU studies aimed to develop sensor systems specifically for detecting early motor delay in infants [45,50]. In a future study, we would like to test the feasibility of implementing the protocol in this paper with IMU sensors. The use of these sensors would allow for the ability to measure and track some kinematic variables of infant movement in a home or clinical setting.

Another promising approach for prediction in cases where multiple factors influence diagnosis, and early detection is desirable, is machine learning. These approaches are well suited, often superior to traditional means-based statistics, for handling the relatively large number of measurements possible from biomechanics [51]. Machine learning approaches have proven successful at detecting upper limb movement patterns from electromyography (EMG) with wearable sensors [52]. They have also proven useful to predict outcomes from CIMT rehabilitation in adults after stroke [53]. Although machine learning approaches have been used to predict the eventual diagnosis of CP based on components of the GMA [54], the specificity of predictions from such models is not at the level desired for widespread adoption [55]. We are not aware of attempts to combine data from various domains, including 3D motion analysis like that presented here, along with observations such as the HINE, TIMP, and GMA, into a machine learning approach for the prediction of an eventual CP diagnosis. Given the relationships evident in a 3D biomechanical analysis from this study, a machine learning approach combining biomechanical measurements with other observational data such as the GMA would seem promising.

## 5. Conclusions

This is a preliminary study with important findings. The exploration of spatiotemporal characteristics of upper extremity movements prior to and during the age of reach onset shows some interesting patterns of change over time, and potential differences between infants with TD and CP. Constraint in real time might increase movement speed and frequency, and reach frequency in infants with CP and TD. Importantly, constraint might increase the movement frequency of an involved arm in real time in infants with CP. Finally, 3D motion capture or wearable sensors might be useful in tracking upper extremity pre-reaching and reaching movements in infants with neuromotor impairment in the upper extremity, such as HCP. Due to the preliminary nature of this study, no recommendations for the immediate application of these results can be made. Further investigation of the hypotheses generated from this study is necessary for the further interpretation of results.

## Figures and Tables

**Table 1:** Overall group mean (standard deviation) of the kinematic variables at 2–6 months of age.

		2 Months	3 Months	4 months	5 Months	6 Months
Movement Length (mm)	CP	62.4 (16.1)	68.6 (19.5)	58.4 (20)	79.8 (15)	52 (13.1)
	NS	62.7 (13.1)	61.4 (16.1)	62.2 (18.4)	87.2 (14.2)	83.9 (16.8)
	TD	64.8 (14.5)	62.2 (13.4)	73.5 (25.9)	79.9 (15.4)	90.2 (33.3)
Path Length (mm)	CP	119 (41.9)	116 (26)	112 (43.6)	138 (29.6)	103 (26.3)
	NS	116 (20.4)	111 (30.9)	113 (38.7)	142 (22.2)	156 (32.1)
	TD	121 (34.9)	113 (20.8)	129 (37.1)	144 (33.7)	155 (47.2)
Straightness Ratio	CP	1.9 (0.3)	1.74 (0.23)	2.09 (0.86)	1.87 (0.33)	2.12 (0.49)
	NS	1.94 (0.3)	1.88 (0.4)	1.91 (0.47)	1.7 (0.22)	2.04 (0.36)
	TD	1.89 (0.31)	1.91 (0.32)	1.9 (0.44)	1.89 (0.31)	1.9 (0.36)
Movement Speed (mm/s)	CP	116 (40.4)	188 (61.4)	111 (55)	244 (130.6)	154 (74.4)
	NS	151 (49.6)	149 (75.6)	130 (67.9)	207 (117.1)	203 (109.4)
	TD	138 (46.8)	174 (72)	203 (136.6)	290 (148)	272 (158.8)
Movement Frequency (#/min)	CP	13.12 (6.94)	24.22 (16.29)	5.25 (3.99)	10.67 (4.88)	6.28 (3.28)
	NS	24.06 (15.14)	14.77 (12.81)	8.44 (6.8)	16.84 (9.61)	17.23 (5.11)
	TD	17.94 (9.27)	14.5 (6.96)	12.93 (9.97)	18.26 (11.94)	17.99 (11.83)
Reach Frequency (#/min)	CP	0.02 (0.07)	1.18 (1.19)	0.22 (0.46)	1.64 (1.52)	0.08 (0.2)
	NS	0.15 (0.32)	0.58 (0.58)	1.17 (1.7)	1.63 (1.82)	3.46 (2.5)
	TD	0.3 (0.66)	1.19 (1.35)	2.06 (3.06)	2.67 (3)	3.63 (3.48)

CP = cerebral palsy, PS = perinatal stroke, TD = typically developing.
